# Enhancing Oxidative Stability of Sunflower Oil during Convective and Microwave Heating Using Grape Seed Extract

**DOI:** 10.3390/ijms13079240

**Published:** 2012-07-24

**Authors:** Mariana-Atena Poiana

**Affiliations:** Faculty of Food Processing Technology, Banat’s University of Agricultural Sciences and Veterinary Medicine from Timisoara, Calea Aradului 119, RO 300645, Timisoara, Romania; E-Mail: atenapoiana@yahoo.com; Tel.: +40-256-277308; Fax: +40-256-277326

**Keywords:** sunflower oil, grape seed extract, primary and secondary oxidation, oxidative stability, convective heating, microwave heating

## Abstract

This study was performed to investigate the effectiveness of grape seed extract (GSE) compared to butylated hydroxytoluene (BHT) on retarding lipid oxidation of sunflower oil subjected to convection and microwave heating up to 240 min under simulated frying conditions. The progress of lipid oxidation was assessed in terms of peroxide value (PV), p-anisidine value (p-AV), conjugated dienes and trienes (CD, CT), inhibition of oil oxidation (IO) and TOTOX value. In addition, total phenolic content (TP) was evaluated in samples before and after heating in order to assess the changes in these compounds relative to the extent of lipid oxidation. The results of this study highlight that GSE showed a significantly inhibitory effect on lipid oxidation during both treatments, although to a different extent. This ability was dose-dependent; therefore, the extent of lipid oxidation was inversely related to GSE level. Convective heating, respective microwave exposure for 240 min of samples supplemented by GSE to a level of 1000 ppm, resulted in significant decreases of investigated indices relative to the control values as follows: PV (48%; 30%), p-AV (29%; 40%), CD (45%; 30%), CT (41%; 36%), TOTOX (35%; 37%). GSE to a level of 600–800 ppm inhibited the lipid oxidation in a similar manner to BHT. These results suggested that GSE can be used as a potential natural extract for improving oxidative stability of sunflower oil during thermal applications.

## 1. Introduction

Sunflower oil is widely used in nutrition as a source of essential linoleic (9-*cis*,12-*cis*-octadecadienoic) acid. Lipid oxidation is the main deterioration process that occurs during thermal processing of vegetable oils containing lipid molecules with polyunsaturation [1,2]. It is well known that edible oils used as cooking medium at high temperatures in the presence of oxygen are subject to thermoxidation, polymerization, and hydrolysis, and the resulting decomposition products not only produce undesirable off-flavors, but can also decrease the nutritional quality of the fried product [3,4]. In addition to convection ovens where heating occurs by forcing hot air to flow around the food, microwave ovens are used in recent times more often for heating, reheating or cooking but the effect of microwave heating on the edible oil can significantly differ from those produced by convective heating. Exposure to microwave determines the increase of free fatty acids level, possible isomerization of the double bonds of fatty acids and oxidation of polyunsaturated fatty acids. As a result, free radicals can be formed in high amounts [5,6].

The addition of synthetic antioxidants for improving oxidative stability of edible oils is discouraged because of their toxicity and carcinogenicity [7]. Recently, there has been increased interest in identifying potential sources in order to obtain natural antioxidants [8–10]. In the last few years attention has been focused on industrial waste, especially those containing residual phenols [11–13]. Grape seeds represent a valuable low-cost raw material for extraction of value-added polyphenolic phytochemicals with potential as food additives or nutraceuticals [12–14]. Grape seeds contain large amounts of phenolic compounds which are responsible for their antioxidant activity, such as: catechin, epicatechin, procyanidins oligomers, procyanidins, phenolic acids that include gallic and ellagic acid, stilbenes such as resveratrol [15–17]. These compounds have many favorable effects on human health, such as inhibiting low-density protein oxidation, decreasing the heart disease risks, and possessing anticarcinogenic properties [18,19]. They also have been proven to be food lipid antioxidants [20]. The mechanism of antioxidative activity of these compounds consists in their capability of radical scavenging, metal chelating, and synergism with other antioxidants [21–23]. Antioxidant activity of GSE has been confirmed by β-carotene linoleate and linoleic acid peroxidation methods [18] as well as by DPPH and phosphomolybdenum complex methods [16]. Most of the studies regarding the GSE effect on lipid oxidation were conducted on meat [24,25]. Mielnik *et al*., [24] demonstrated that GSE was effective in inhibiting lipid oxidation of cooked turkey meat during storage. Brannan *et al.*, [25] reported that GSE inhibited lipid oxidation in muscle during refrigerated and frozen storage. Rababah *et al.*, [26] proved that GSE is an effective antioxidant to minimize lipid oxidation in corn chips during storage. The information about GSE effectiveness in delaying the oil quality deterioration during applications which require heating to high temperatures seems to be limited. Shaker *et al.*, [27] reported that GSE (200 ppm) exhibited reasonable antioxidant activity during the first day of sunflower oil heating at 60 °C but showed pro-oxidative effect with prolonged treatment. Jayaprakasha *et al*., [17] reported that GSE exhibited high antioxidant activity, and may be used for food preservation and health supplements.

In order to exploit the efficiency of GSE as a natural antioxidant on retarding the lipid oxidation process development in sunflower oil subjected to heating, this new attempt was undertaken. This work was conducted in order to assess the effectiveness of natural antioxidant (GSE) compared to synthetic antioxidant (BHT) towards lipid oxidation in the heating time of sunflower oil. For this purpose, two heating processes (in electrical convection oven and microwave oven) were analyzed as a function of time, antioxidants (BHT,GSE) as well as antioxidant levels. The progress of lipid oxidation was monitored by chemical indices: PV, p-AV, CD and CT as well as TOTOX value and IO. In addition, total phenolic content (TP) was evaluated in samples before and after heating in order to assess the changes of these compounds relative to the extent of lipid oxidation Also, for samples supplemented by GSE at the 1000 ppm level, TP in the heating time was monitored, relative to the progress of oxidative lipid degradation.

### 2. Results and Discussion

The present study was carried out in refined sunflower oil, free of additives, supplemented by five concentration levels for the GSE (*i.e.*, 200, 400, 600, 800 and 1000 ppm) or one level of BHT (200 ppm). Oil samples were subjected to convective and microwave heating up to 240 min under simulated frying conditions, at comparable temperatures. The doses of GSE were chosen in agreement with previous studies that have proved that the inhibitory effect on lipid oxidation increased with the antioxidant concentration [19,24–26]. In order to highlight the effect of BHT and GSE on the investigated indices, experimental data were processed by one-way ANOVA test. Based on information obtained by statistical processing, the significance of changes in monitored indices appears to be a response to BHT and GSE addition in the heating time.

### 2.1. Evaluation of Antioxidant Properties of GSE

A number of studies that focused on the antioxidant activity of bioactive compounds of grape seeds, have reported variable TP content for GSE ranging from 580–3930 μmol gallic acid/g, possibly due to differences in grape varieties and/or in extraction methods and conditions [22,28]. Polyphenols in grape seeds are mainly gallic and ellagic acid, stilbenes, flavonoids such as catechin, epicatechin, procyanidins and anthocyanins [15,16]. In this study, TP content of GSE processed from the wine grape variety “Merlot Recas” was 1019.83 μmol GAE/g extract, Table 1. This value is about 28% higher than those reported by Alexa *et al.*, [29] in which GSE was obtained from another Romanian wine grape variety (Cabernet Sauvignon, Recas winery).

The reducing power of BHT and GSE is a reliable indicator of their antioxidant activity, indicating that the antioxidant compounds are electron donors and can reduce the oxidized intermediates of the lipid peroxidation process [17,19]. In agreement with results reported by Bonilla *et al.*, [20], GSE presented lower antioxidant activity than the synthetic antioxidants—BHT; see Table 1. The composition of phenolic compounds is likely to be more important than the antioxidant activity [27,28]. Phenolic compounds are known to act as antioxidants not only due to their ability to donate hydrogen or electron, but also attributed to their stable radical intermediates, which prevent the oxidation of various food ingredients particularly fatty acids and oil [9]. The antioxidant activity may vary widely depending on the lipid substrate. Hydrophilic antioxidants are more effective in lipid systems, whereas lipophilic antioxidants work better in emulsions where more water is present. In lipophilic environment, hydrophilic antioxidants are oriented to oil-air interface, and provide better protection against lipid oxidation than in hydrophilic environment where hydrophilic antioxidants prefer to dilute and thus act poorly against lipid oxidation [30].

### 2.2. Impact of Supplementation with GSE and BHT on Oil Quality in the Heating Time

#### 2.2.1. Peroxide Value (PV) and Inhibition of Oil Oxidation (IO)

PV and IO were used as indicators for the primary oxidation of sunflower oil. Hydroperoxides are the primary products of lipid oxidation. They are odorless and colorless, but are labile species that can undergo both enzymatic and non-enzymatic degradation to produce a complex array of secondary products. Determination of peroxides can be used as oxidation index for the early stages of lipid oxidation [4,9]. Measuring the content of primary oxidation products is limited due to the transitory nature of peroxides, but their presence may indicate a potential for later formation of sensorial objectionable compounds. PV increases only when the rate of peroxides formation exceeds that of its destruction. Data from Table 2 express the changes of PV recorded in the heating time in response to oil supplementation with BHT and GSE.

According to these results it can be observed that thermal treatments promoted oxidation in sunflower oil leading to a significant increase in PV but this effect was markedly reduced by supplementation with GSE and BHT. At any time of convective and microwave heating, significant differences (*p* < 0.05) in PV were observed between the control sample and oil samples with BHT (200 ppm) or supplemented by various doses of GSE. The inhibitory effect of GSE against primary oxidation of lipid was concentration-dependent. These results are consistent with data reported previously by Brannan *et al.*, [25], Rababah *et al.*, [26], Shaker *et al*., [27] and show that the antioxidant compound of GSE have an important role in inhibiting of free radical formations during the initiation step of oxidation, interruption of the propagation of the free radical chain reaction by acting as an electron donor, or scavengers of free radicals in sample. At the end of convective heating, PV of samples with BHT decreased by approx. 32% relative to the control while in samples with different levels of GSE, PV decreased in the range 19–48% relative to the control.

PV showed significant changes (*p* < 0.05) during microwave heating up to 240 min, but the values did not increase constantly in the heating time, Table 2. Che Man *et al*., [31] reported a decrease in PV of oil samples after an initial increase. PV still tends to increase during the early stages of oxidation, when the rate of hydroperoxides formation is higher than the rate of their decomposition. A significant decrease of PV after an initial increase confirms that peroxides formed in the early stages of oxidation are unstable and highly susceptible to further changes that result in the formation of secondary products of oxidation [32]. Probably, the hydroperoxides accumulated in the initial stage of heating were decomposed due to higher temperature. However, a low PV represents either early or advanced oxidation. At the end of heating in microwave oven, PV for samples with BHT decreased by approx. 25% relative to the control and in the range 4–30% relative to the control for samples supplemented by various doses of GSE.

**Figure 1:**
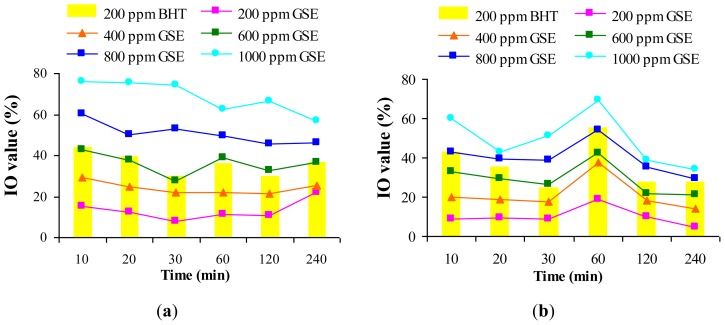
Inhibitory effect of GSE and BHT on primary lipid oxidation during oil heating (**a**) in convection oven; (**b**) in microwave oven.

Figure 1 provides information about the inhibitory power of GSE and BHT on primary lipid oxidation in the heating time. Based on statistical test, it could be concluded that at any time of heating treatments the increasing of GSE level resulted in significant increases of IO (*p* < 0.05). The results indicated that GSE to a level of 200–400 ppm had an inhibition power lower than BHT during both heating techniques.

During convective heating GSE at the 600 ppm level had an inhibitory effect of primary lipid oxidation comparable to BHT, while in the microwave heating time inhibitory effect of BHT was similar to GSE at the 800 ppm level. GSE to a level of 1000 ppm showed an inhibition power higher than BHT in both treatments. GSE did not show pro-oxidative effect during both treatments up to 240 min. According to Shaker *et al.*, [27] the pro-oxidative effect had been proven by increasing the amount of oxidized products with prolonged heating, when additives were added.

#### 2.2.2. p-Anisidine Value (p-AV)

During lipid oxidation, hydroperoxides, the primary reaction products, decompose to produce secondary oxidation products (aliphatic aldehydes, ketones, alcohols, acids and hydrocarbons) which are more stable during the heating process, responsible for off-flavors and off-odors of edible oils. In order to ensure a better monitoring of lipid oxidation process in the heating time, the simultaneous detection of primary and secondary lipid oxidation products is necessary. p-AV is a reliable measurement of the amount of secondary oxidation products [9,33].

Table 3 presents the changes recorded in p-AV in the heating time as effected by supplementation with GSE and BHT. It can be observed that convection and microwave heating promoted rapid transformation to secondary products which contributes to the off-flavors of sunflower oil. Addition of BHT and various levels of GSE resulted in significant decrease in p-AV (*p* < 0.05) relative to the control sample. The highest level of GSE provides the best protection against secondary oxidation of oil samples subjected to heating. These data are in agreement with those reported by Kalantzakis and Blekas [10] which highlight that the natural extracts showed a significant inhibitory effect against thermal oxidation of refined oils heated at 180 °C.

At the end of convective heating, p-AV of samples with BHT decreased by approx. 16% relative to the control, while addition of GSE resulted in various decreases in p-AV depending on the dose, in the range 10–29% relative to the control. Also, data presented in Table 3 revealed that after 240 min of microwave heating, p-AV of samples mixed with BHT decreased by approx. 26% relative to the control and in the range 10–40% relative to the control for oil samples with various doses of GSE. From these results it can be seen that polyphenolic compounds found in GSE had a strong inhibitory effect on the secondary lipid oxidation. From the statistical test it could be concluded that, for both treatments, the extent of secondary lipid oxidation, was significantly decreased by increasing of GSE dose added in sunflower oil (*p* < 0.05). At the end of heating, there were no significant differences (*p* > 0.05) between sunflower oil samples treated with BHT and those samples treated with GSE to a level of 600 ppm. This means that GSE to a level of 600 ppm provided protection in a similar manner to BHT against secondary lipid oxidation. GSE to a level over 800 ppm induced an inhibitory activity higher than BHT, while GSE to a level of 200–400 ppm had a lower potential to inhibit the secondary oxidation than BHT.

#### 2.2.3. Total Oxidation Value (TOTOX Value)

**Figure 2:**
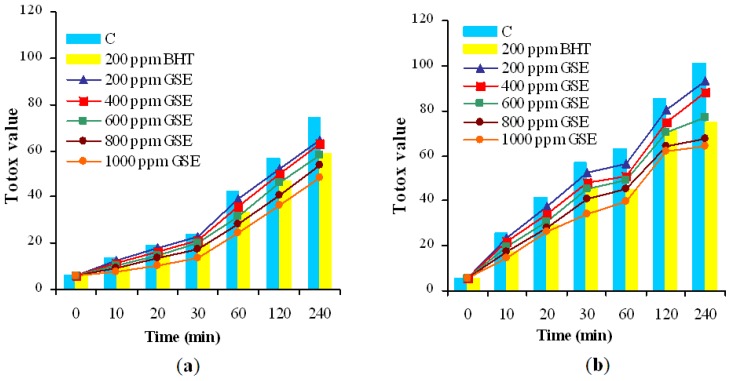
Impact of GSE and BHT on TOTOX value during sunflower oil heating (**a**) in convection oven; (**b**) in microwave oven.

The use of PV and p-AV together provides a comprehensive overview of the oxidation process in oils. This is a mathematical prediction of oxidative stability and the value is calculated as TOTOX value. TOTOX value was used as an indication of overall oxidative stability and was correlated with the extent of oil deterioration [33]. TOTOX value for oil samples subjected to treatments in convection and microwave oven markedly increased with increasing heating time, see Figure 2.

TOTOX values for samples mixed with BHT and GSE were significantly lower than the value registered for control (*p* < 0.05). After 240 min of convective heating, oil supplementation with various doses of GSE resulted in decrease of TOTOX value in the range 13–35% relative to the control while exposure to microwave resulted in decline of TOTOX value in the range 8–37% relative to the control. The highest level of GSE had the best inhibitory effect on oil oxidation in the heating time. At any stage of both treatments, the lowest TOTOX values by supplementation with GSE to a level of 1000 ppm were recorded. Also, GSE to a level of 600 ppm inhibited the lipid oxidation in a similar manner to BHT.

Although, there is sufficient information available about the consequences of microwave heating on the composition and nutritional quality of food, little has been published on the changes in oxidative stability of sunflower oil supplemented by natural extract as an effect of microwave exposure. Also, there is no data on the inhibitory effect of GSE against lipid oxidation in sunflower oil during microwave heating. This effect was assessed in this study in parallel with convective heating under comparable temperatures. On this topic, there was controversy regarding free radical formation when oils and fatty food are subjected to microwave treatment [6,34,35]. Based on Figure 2, it can be seen that oxidative degradation was greater in the microwave heating time than in the convective heating at comparable temperatures. Data obtained in this study are in agreement with others reported by Dostalova *et al.* [6], Megahed [34], Erkan *et al.* [35] that concluded that even a short period of microwave heating accelerates the formation of some undesirable and harmful compounds (e.g. oxidation products, transformed pigments) due to interactions between electromagnetic field with the chemical constituents of oil. These results reveal that, in the sunflower oil system, GSE could limit the lipid oxidation developed in the heating time. Probably, the addition of natural extract created an oil system surrounded by antioxidants that were able to prevent oxidation because phenolic compounds were located on the interface of the lipid system. The enhancement of oxidative stability of sunflower oil by supplementation with GSE was dose-dependent.

#### 2.2.4. Conjugated Dienes (CD) and Trienes (CT)

The polyunsaturated fatty acids oxidation occurs with the formation of hydroperoxides. Immediately after peroxides have been formed, the non-conjugated double bonds present in natural unsaturated lipids suffer a rearrangement generating conjugated dienes (CD), which absorb at 232 nm [1]. When polyunsaturated fatty acids containing three or more double bonds (e.g., linolenic acid) undergo oxidation, the conjugation can be extended to include another double bond resulting in the formation of conjugated trienes (CT) which absorb at 268 nm. The changes in UV absorbance at 232 and 268 nm, quantified by K 232 and K 268 have been used as a relative measure of oxidation [5,33]. The presence of CD and CT is a better measurement of oxidation because they remain in the frying oil [36]. In accordance with this, the instability of peroxide molecules may also explain the decrease in PV during advanced stages of rancidity, so that breakdown into smaller molecules compounds associated with oxidation of lipids would be expected to occur. The increase in K 232 and K 268 is proportional to the uptake of oxygen and formation of peroxides during the early stages of oxidation as well as with the degradation rate of linoleic acid [31,36].

**Figure 3:**
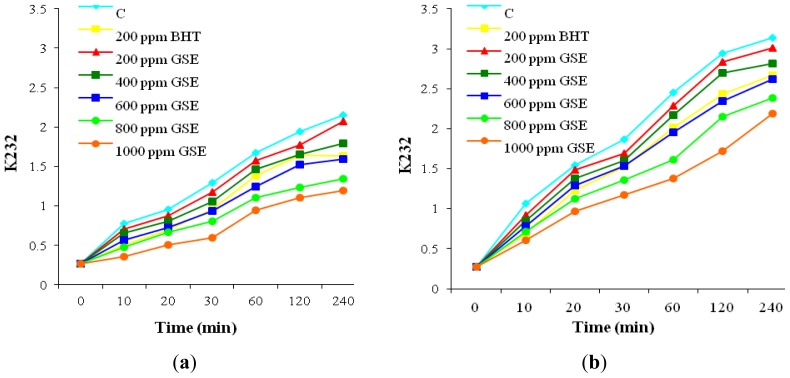
Effect of supplementation with GSE and BHT on K 232 during oil heating (**a**) in convection oven; (**b**) in microwave oven.

**Figure 4:**
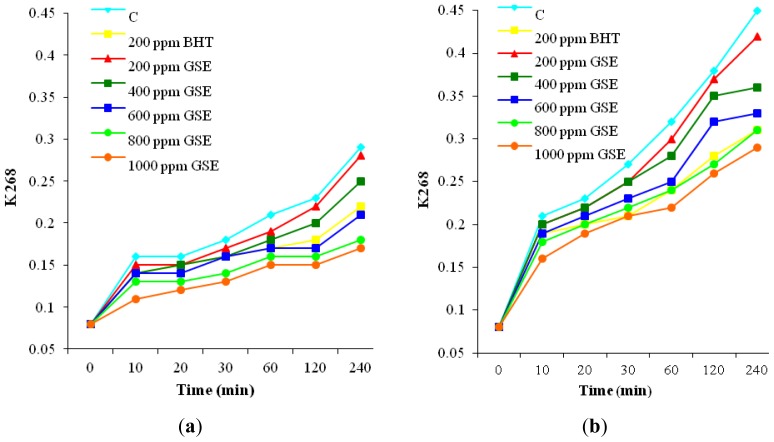
Effect of supplementation with GSE and BHT on K 268 during oil heating (**a**) in convection oven; (**b**) in microwave oven.

The results presented in Figures 3 and 4 highlight that both heating processes caused positional rearrangement of the double bonds in sunflower oil and, consequently, a part of the non-conjugated system was converted to conjugated diene and triene double bonds.

Accordingly, the absorbance values of these molecules were gradually increased with the increase in the heating time. Based on Figure 3 it can be noticed that the rate of CD formation was higher than the decomposition rate, leading to their accumulation in oil. The values registered for CD are a measure of lipid alterations due to double bonds conjugation as a consequence of primary oxidation. In Figure 4 the changes of K 268 are shown, associated with CT accumulation during heating. These changes reflect the appearance of oxidation by-products such as unsaturated α- and β-diketones and β-ketones, typical of oils in the process of going rancid [31]. As in the previous case, CT level increased with heating period. By oil supplementation with BHT and GSE the accumulation of CD and CT decreased. The inhibitory effect of GSE on CD and CT formation was dose-dependent. The oil samples supplemented by the highest dose of GSE had the lowest amounts of CD and CT at any stage of heating.

The inhibition of CD and CT by addition of GSE is important in the early stages of lipid peroxidation reactions, in order to prevent the subsequent formation of reactive lipid radicals. The antioxidant ability of GSE to reduced CD and CT accumulation was higher in the convective heating than in the microwave treatment. At the end of treatment, GSE at level of 1000 ppm reduced CD accumulation by approximately 45% relative to the control in the convective heating and by 30% relative to the control in the microwave treatment. Also, by supplementation with GSE at the 1000 ppm level, CT accumulation decreases by 41% relative to the control in the convective heating and by 36% relative to the control during microwave heating.

These data prove the efficiency of natural antioxidants derived from grape seeds in slowing down lipid degradation and increasing oxidative stability of oil even when exposed to high temperatures. In both heating methods, GSE at level of 600 ppm showed the same effectiveness in reducing the accumulation of CD as BHT. Also, BHT inhibited the formation of CT in a similar manner to GSE at the 600 ppm level during convective heating. Less active against CT formation was GSE in samples subjected to microwave heating. Only a level of 800 ppm GSE provided a similar protection as BHT. These results are consistent with those reported by El Anany [2] and Rehab [4] which revealed that the addition of natural extracts to sunflower oil heated at 180 ± 5° induced a strong antioxidant activity and at a level of 800 ppm were superior to BHT in increasing oil stability.

### 2.3. Correlations

**Figure 5:**
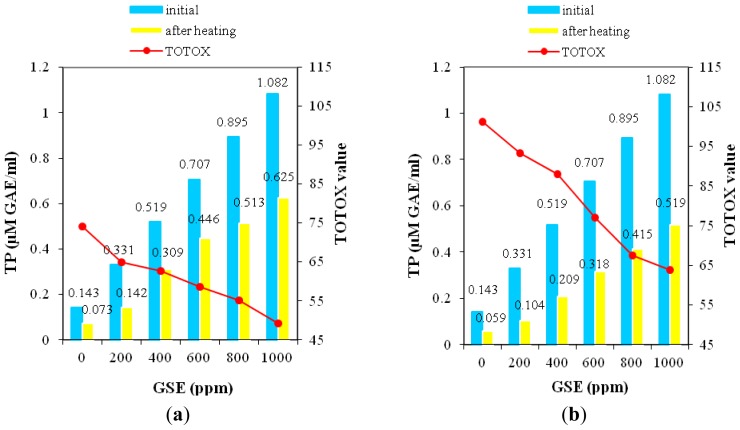
Heating impact on phenolic content (TP) in sunflower oil supplemented by GSE related to TOTOX value (**a**) convective heating; (**b**) microwave heating.

Figure 5 provides information about TP content in oil samples supplemented by various levels of GSE at the beginning and at the end of treatment. Based on recorded differences the alterations in these compounds is a direct effect of heating. The declines recorded in TP content as a result of convective heating for 240 min were in the range 42–57% of the initial value, while by microwave heating the losses of TP content were in the range 52–69% relative to the initial value. The extent of lipid oxidation was greater in samples treated in a microwave oven than in those subjected to convective heating; consequently, in order to inhibit the lipid oxidation larger amounts of TP were required in samples exposed to microwave than in those heated by convection.

Based on TOTOX value, it can be seen that in oil samples supplemented by GSE at the 1000 ppm level the lowest extent of lipid oxidation was recorded after heating. These data highlight that TP compounds significantly contributed to antioxidant activity of GSE in the heating times recorded. Studies on correlations between antioxidant activity and TP content of GSE have previously been reported [22,24]. These results are in agreement with findings by other authors Mielnik *et al.*, [24] who reported strong linear relationships between the amount of antioxidants and the ability of GSE to prevent lipid oxidation, indicating that the antioxidants efficiency is dose-dependent.

**Figure 6:**
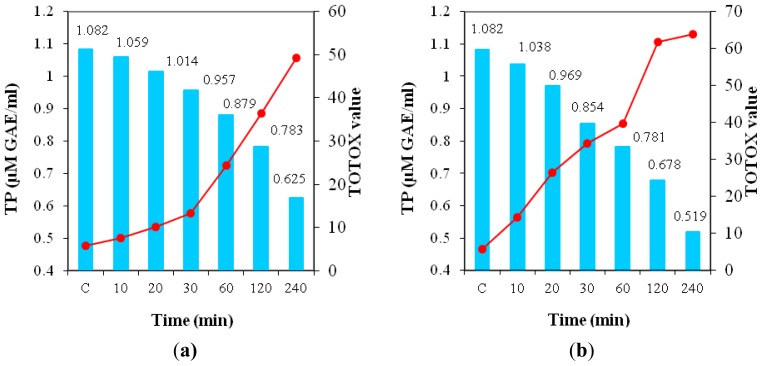
Alterations of TP in oil supplemented by GSE (1000 ppm) related to TOTOX value in the heating time (**a**) in convection oven; (**b**) in microwave oven.

For samples supplemented by GSE at the 1000 ppm level, Figure 6 shows the changes in TP content as a function of time related to TOTOX value. It is worth noting that both heating treatments resulted in severe losses in TP content but microwave exposure caused a more advanced degradation of these compounds than convection heating. The extent of TP degradation increased with heating time, thus in the first 20 min, both treatments of oil samples caused only minor losses in TP (6–10% of the initial value), and the lipid oxidation was reduced. Significant losses of TP were detected only after 120 min of heating, when there was an advanced lipid oxidation.

These data highlight that the lowest content of TP are found in samples with the highest extent of oxidative deterioration expressed by the highest TOTOX value. Also, a high negative correlation was detected between TOTOX value and TP during heating (*r* = −0.994 for convective heating and *r* = −0.973 for microwave treatment). Data obtained in this study are consistent with results reported by Chantzos and Georgiou [37] and strengthen the concept that total antioxidant capacity is inversely related to the extent of lipid oxidation as assessed through the TOTOX value that is considered to be an appropriate physico-chemical index for monitoring edible oil oxidation. In this study, a high positive correlation was detected between IO values and TP content consumed as an effect of oxidative degradation by heating (*r* > 0.97), Table 4.

A significant negative correlation was found between TOTOX values and TP consumed in the heating time (*r* > 0.98), Table 4. This could be attributed to the protective action of TP against thermo-oxidative degradation. Also, high negative correlation coefficients (*r* > 0.95) were found between TP consumed in the heating time and PV, p-AV, CD and CT, demonstrating once again that the efficiency of GSE on retarding lipid oxidation was concentration-dependent. The composition of TP compounds is more important than the TP concentration [28]. Further research is needed in order to obtain more reliable results on the determination of chemical composition of GSE and to elucidate the compounds that contributed to the strong inhibitory effect exhibited by GSE on lipid oxidation in the heating time.

## 3. Experimental Section

### 3.1. Processing of GSE

Pressed grape pomace obtained from the Romanian grape variety Merlot (*Vitis vinifera* L.) was taken from Recas winery (western part of Romania, vintage 2010). Seeds conditioning were performed according to Alexa *et al.*, [29]. Thus, grape seeds were manually removed from the skin and pulp and dried at 60 °C for 24 h in a drying oven (Binder, Germany) then ground using a grinder (Grindomix Retsch GM 2000) and passed through a 60 mesh. The ground seeds were defatted in an automated extractor (Velp Scientifica, Italy) using hexane (1:5) for 2 h. The defatted residue was air dried overnight to remove residual hexane. In the view of bioactive compounds extraction from grape seeds were taken into account previous results on this topic reported by Yilmaz and Toledo [16] and Lafka *et al.*, [18]. 50 g of defatted seeds powder were mixed with 1000 mL ethanol 70% (v/v), kept in the shaking incubator at 25 °C for 48 h and filtered in vacuum then centrifuged (4500 rpm, 15 min). The obtained supernatants were evaporated until 100 mL under reduced pressure at 50 °C using a rotary evaporator (Heidolph Laborota 4000). The ethanolic extract was freeze-dried using a lyophilizer (Ilshin Lab Co., Ltd.) and the obtained powder (GSE) were kept frozen (−18 °C) until the analyses were performed.

### 3.2. Antioxidant Activity (FRAP Assay)

The antioxidant activity of GSE and BHT was measured using the ferric reducing antioxidant power (FRAP) assay [38]. In order to evaluate antioxidant activity, 0.1 g GSE, respectively BHT were mixed with 20 mL ethanol/water (70:30, v/v) for 10 min, then the solution was filtered using a Buckner funnel and Whatman No 1 filter paper and each filtrate was used for analysis. Ferric to ferrous ion reduction at low pH (3.6 in acetate buffer) produces a colored ferrous-tripyridyltriazine complex. FRAP values are obtained by reading the absorbance changes at 595 nm using a UV-VIS spectrophotometer (Analytic Jena Specord 205), which are linear over a wide concentration range. FRAP values were expressed as μM Fe^2+^ equivalents/g GSE, respectively BHT. All determinations were performed in triplicate.

### 3.3. Total Phenols Assay

Total phenolic content (TP) of GSE and oil samples was determined using the Folin-Ciocalteu colorimetric method [39,40]. In order to extract total phenolic compounds from sunflower oil, 2 mL oil was mixed with 20 mL ethanol/water (70:30, v/v) by sonication at room temperature for 30 min. The mixture was centrifuged (5000 rpm, 10 min) at room temperature and the supernatant was used for TP analysis. TP content of GSE was determined using the extract obtained previously for assessing antioxidant capacity. A calibration curve using gallic acid was prepared and the absorbance of the standards and samples were measured at 750 nm. Results were expressed as μM gallic acid equivalents (GAE) per g GSE, respectively per mL oil (when calculating TP content in oil samples). All determinations were performed in triplicate.

### 3.4. Application of GSE to Sunflower Oil

Freshly refined sunflower oil (7 kg), free of synthetic antioxidants, was divided into 7 portions. Five of them, were supplemented with 200, 400, 600, 800 and 1000 ppm GSE, the sixth portion was mixed with synthetic antioxidant BHT at their legal limit of 200 ppm and the last portion, without antioxidant, was used as a control. Before applying, GSE and BHT were separately mixed with a minimum amount of ethanol 96% (v/v) (to ensure of dispersion in oil) in an ultrasonic water bath and then were added to sunflower oil, mixed for 10 min and vacuum evaporated. Control sample was prepared using the same amount of ethanol 96% (v/v) used to dissolve BHA and GSE [41].

### 3.5. Heating Processes

In order to explore the effect of microwave and convection heating on refined sunflower oil supplemented by GSE and BHT, samples were separately heated under simulated frying conditions using a convection electric oven and a microwave oven. Experimental design of heating processes applied was developed in agreement with previously studies on this topic [5,6,42]. Preliminary tests were carried out in order to ensure comparable temperature regimes during both treatments.

#### 3.5.1. Convective Heating

Oil samples (25.0 ± 0.5 g) were weighed into the Pyrex Petri dish with inner diameter 11 cm and placed alone in the electrical convection oven (Esmach, Italy, 1200W, 50Hz) regulated at 200 °C. Samples were separately heated for 10, 20, 30, 60, 120 and 240 min. The temperatures of the oil samples immediately after each heating period were determined by inserting a calibrated chromel-alumel thermocouple (HI 935009, Hanna Instruments) into the oil samples. Table 5 shows the internal temperatures of oil samples heated convective and by microwaves. After each heating period, the samples were taken out of the oven cooled rapidly and stored at −18 °C till the analysis. Separate samples were used for different heating times. After each heating period the oven was stopped for 30 min and the oven door was opened in order to be cooled down before starting the next heating.

#### 3.5.2. Microwave Heating

Oil samples (25.0 ± 0.5 g) were weighed into the Pyrex Petri dish with inner diameter 11 cm and placed alone in the center of the rotating plate (31.5 cm in diameter) of the microwave oven for home appliances (Candy, Model CMG 2394DS, 50 Hz, microwave frequency 2450 MHz, maximum power 900 W). The samples were heated at the input of 80% power (720 W) for the same periods referred to convective heat. The oil temperatures after each heating time were performed similarly to previous heating. Both heating treatments were performed at comparable temperatures: after 30 min from the starting treatment, the oil temperature remained at 185 ± 7 °C during the whole monitored period, Table 5. After each heating time, the samples were taken out of the oven, cooled rapidly at room temperature and stored in sealed tubes at −18 °C until analyzed. Between tests, the oven door was opened for 30 min to facilitate the cooling process.

### 3.6. Evaluation of Lipid Oxidation

The progress of lipid oxidation was monitored by measuring of standard chemical indices, respective peroxide value, p-anisidine value and conjugated dienes and trienes. Besides these indices, total oxidation and inhibition of the oil oxidation were evaluated.

*The peroxide value (PV*) was determined iodometrically according to standard methods for the oils analysis and the results were expressed in meq/kg oil [43,44].

*The inhibition of oil oxidation (IO*) was calculated according to the formula (1) [41]:

(1) $$\text{IO}(\%)=(1-\frac{\text{PV increase of sample}}{\text{PV increase of control}})\times 100$$

*The p-anisidine value (p-AV*) is a measurement of carbonyl content in the oils or fats, and was determined by the standard method according to AOCS [43]. It is based on the reactiveness of the aldehyde carbonyl bond on the p-anisidine amine group, leading to the formation of a Schiff base that absorbs at 350 nm. 2 g of the sunflower oil samples were dissolved in 25 mL isooctane and absorbance (A_1_) of this fat solution was measured at 350 nm against a blank of isooctane. An aliquot (5 mL) of this solution, respectively 5 mL of isooctane (as blank) was transferred to each of two test tubes of 10 mL and 1 mL anisidine solution (0.25% g/v glacial acetic acid) was added to each. After 10 min, the absorbance (A_2_) was measured at 350 nm against isooctane containing p-anisidine. P-AV was calculated according to the formula (2):

(2) $$\text{p}-\text{AV}=25\times \frac{1.2\times {\text{A}}_{2}-{\text{A}}_{1}}{\text{w}}$$

*The total oxidation value (TOTOX*) was used to estimate the oxidative deterioration of lipids. TOTOX value is defined as the sum of both values (PV and p-AV) to total oxidation and was calculated according to the formula (3) [33]:

(3) TOTOX value=2×PV+p-AV

*The conjugated dienes (CD*) and *conjugated trienes hydroperoxides (CT*) formed were measured in the lipids, according to the method reported by Kim and Labella [45]. Oil samples (approx. 200 mg) was dissolved in 25 mL isooctane and mixed thoroughly. The absorbance values were measured in UV region at 232 and 268 nm for CD and CT, respectively. The results are expressed as the specific extinction values K 232 and K 268.

### 3.7. Statistical Analysis

All determinations were carried out in triplicates and values were expressed as means ± standard deviation (SD). Significant statistical differences of investigated parameters were determined by Fisher’s least significant differences (LSD) test at *p* < 0.05, after analysis of variance (ANOVA one-way). Data were analyzed by ANOVA to ascertain if the GSE levels or BHT were a source of variance related to measured parameters. Computations Tukey post-hoc means comparisons and Levene’s test for equal variance was also included. Statistical processing data was performed using the Statistical Analysis System-SAS (Software version 8.1; SAS Institute, Inc.: Cary, NC, USA, 2000).

## 4. Conclusions

The analysis of convective and microwave heating of sunflower oil, supplemented by various levels of GSE in terms of standard chemical indices, appears to be a valuable tool to assess the ability of this natural extract to inhibit lipid oxidation. Exposing the oil samples to convective and microwave heating caused the formation of hydroperoxides and secondary oxidation products resulting in significant alterations to sunflower oil quality. Supplementation with GSE and BHT prior to heating significantly improved oxidative stability of sunflower oil. The efficiency of GSE to enhance the oxidative stability of sunflower oil during investigated thermal applications increased with increasing antioxidant concentration in the studied range (200–1000 ppm). Oil supplementation with GSE to a level in the range 600–800 ppm inhibited the lipid oxidation in a similar manner to BHT, while a level over 800 ppm limits thermo-oxidative degradation of sunflower oil more than BHT. Also, these results highlight that TP content of samples could be correlated to oxidative deterioration strengthening the concept that total antioxidant capacity is inversely related to the extent of lipid oxidation in the heating time range. These data prove that GSE is a very effective inhibitor of lipid oxidation in applications which require oil heated at high temperatures and can be recommended as a potential natural antioxidant for the edible oil industry.

## Figures and Tables

**Table 1:** Antioxidant characteristics of grape seed extract (GSE) and butylated hydroxytoluene (BHT).

Sample	FRAP value (μmol Fe^2+^/g)	Total phenolics (μmol GAE/g)
GSE	1231.56 ± 17.29	1019.83 ± 15.68
BHT	1339.14 ± 24.93	-

**Table 2:** Effect of GSE and BHT on peroxide value (PV) during sunflower oil heating (**a**) in convection oven; (**b**) in microwave oven.

Time (min)	PV (meq/kg oil)						

	Control	BHT 200 ppm	GSE (ppm)				

			200	400	600	800	1000
**(a) Convective heating**							

0	1.77 ± 0.09 ^a^	1.77 ± 0.09 ^a^	1.77 ± 0.09 ^a^	1.77 ± 0.09 ^a^	1.77 ± 0.09 ^a^	1.77 ± 0.09 ^a^	1.77 ± 0.09 ^a^
10	4.14 ± 0.12 ^a^	3.09 ± 0.10 ^c^	3.78 ± 0.11 ^b^	3.45 ± 0.18 ^b^	3.13 ± 0.14 ^c^	2.72 ± 0.13^e^	2.34 ± 0.12 ^f^
20	4.60 ± 0.16 ^a^	3.47 ± 0.18 ^c^	4.23 ± 0.18 ^a^	3.89 ± 0.17 ^b^	3.53 ± 0.12 ^c^	3.18 ± 0.11 ^d^	2.46 ± 0.08 ^e^
30	5.37 ± 0.16 ^a^	4.28 ± 0.13 ^c^	5.08 ± 0.21 ^a^	4.61 ± 0.23 ^b^	4.37 ± 0.24 ^c^	3.47 ± 0.17 ^d^	2.69 ± 0.18 ^e^
60	8.89 ± 0.35 ^a^	6.30 ± 0.24 ^c^	8.09 ± 0.19 ^b^	7.34 ± 0.27 ^b^	6.13 ± 0.23 ^c^	5.38 ± 0.26 ^d^	4.47 ± 0.33 ^e^
120	10.01 ± 0.41 ^a^	7.48 ± 0.26 ^c^	9.12 ± 0.25 ^b^	8.25 ± 0.23 ^b^	7.31 ± 0.33 ^d^	6.24 ± 0.24 ^e^	5.19 ± 0.32 ^f^
240	12.05 ± 0.76 ^a^	8.24 ± 0.31 ^c^	9.81 ± 0.50 ^b^	9.43 ± 0.59 ^b^	8.29 ± 0.23 ^c^	7.31 ± 0.27 ^d^	6.22 ± 0.32 ^e^

**(b) Microwave heating**							

0	1.77 ± 0.09 ^a^	1.77 ± 0.09 ^a^	1.77 ± 0.09 ^a^	1.77 ± 0.09 ^a^	1.77 ± 0.09 ^a^	1.77 ± 0.09 ^a^	1.77 ± 0.09 ^a^
10	9.69 ± 0.45 ^a^	6.27 ± 0.37 ^d^	9.00 ± 0.43 ^a^	8.13 ± 0.46 ^b^	7.12 ± 0.55 ^c^	6.27 ± 0.35 ^d^	4.79 ± 0.31 ^e^
20	14.73 ± 0.40 ^a^	10.08 ± 0.47 ^d^	13.5 ± 0.39 ^b^	12.3 ± 0.35 ^c^	10.96 ± 0.37 ^d^	9.61 ± 0.20 ^e^	7.09 ± 0.34 ^f^
30	19.14 ± 0.61 ^a^	14.71 ± 0.42 ^d^	17.59 ± 0.61 ^b^	16.1 ± 0.34 ^c^	14.57 ± 0.36 ^d^	12.36 ± 0.45 ^e^	9.88 ± 0.39 ^f^
60	15.02 ± 0.55 ^a^	7.59 ± 0.46 ^d^	12.52 ± 0.41 ^b^	10.04 ± 0.69 ^c^	9.38 ± 0.43 ^c^	7.88 ± 0.53 ^d^	5.79 ± 0.45 ^e^
120	18.81 ± 0.37 ^a^	14.02 ± 0.38 ^d^	17.07 ± 0.49 ^b^	15.7 ± 0.51 ^c^	15.09 ± 0.50 ^c^	12.83 ± 0.55 ^e^	12.24 ± 0.34 ^f^
240	16.21 ± 0.38 ^a^	12.13 ± 0.34 ^c^	15.56 ± 0.40 ^a^	14.17 ± 0.58 ^b^	13.15 ± 0.33 ^b^	11.97 ± 0.42 ^c^	11.28 ± 0.17 ^d^

Means in a row (a-f across GSE level) followed by the same letter are not significantly different (*p* < 0.05).

**Table 3:** Effect of GSE and BHT on p-AV during sunflower oil heating (**a**) in convection oven; (**b**) in microwave oven.

Time (min)	p-AV						

	Control	BHT 200 ppm	GSE (ppm)				

			200	400	600	800	1000
**(a) Convective heating**							

0	2.28 ± 0.16 ^a^	2.28 ± 0.16 ^a^	2.28 ± 0.16 ^a^	2.28 ± 0.16 ^a^	2.28 ± 0.16 ^a^	2.28 ± 0.16 ^a^	2.28 ± 0.16 ^a^
10	5.27 ± 0.43 ^a^	3.68 ± 0.31 ^c^	4.95 ± 0.38 ^a^	4.56 ± 0.31 ^a^	4.08 ± 0.36 ^b^	3.77 ± 0.32 ^c^	2.81 ± 0.26 ^d^
20	9.91 ± 0.54^a^	7.47 ± 0.52 ^c^	9.48 ± 0.61 ^a^	8.34 ± 0.69 ^b^	7.85 ± 0.51 ^c^	7.39 ± 0.56 ^c^	5.24 ± 0.41 ^d^
30	13.3 ± 0.45^a^	11.23 ± 0.71 ^b^	12.79 ± 0.71 ^a^	12.27 ± 0.79 ^a^	11.51 ± 0.65 ^a^	10.24 ± 0.70 ^c^	8.03 ± 0.50 ^d^
60	24.95 ± 1.07 ^a^	20.81 ± 1.01 ^b^	23.01 ± 1.04 ^a^	21.7 ± 1.29 ^b^	19.79 ± 1.14 ^c^	17.79 ± 1.08 ^d^	15.89 ± 1.06 ^e^
120	36.24 ± 1.22 ^a^	31.82 ± 1.36 ^b^	34.01 ± 1.21 ^a^	33.28 ± 1.13 ^a^	31.39 ± 1.33 ^b^	28.48 ± 1.64 ^c^	26.04 ± 1.66 ^d^
240	50.03 ± 2.01 ^a^	42.16 ± 1.67 ^c^	45.25 ± 1.86 ^b^	43.9 ± 2.14 ^c^	41.73 ± 1.81 ^c^	39.18 ± 1.62 ^d^	35.75 ± 1.44 ^f^

**(b) Microwave heating**							

0	2.28 ± 0.16 ^a^	2.28 ± 0.16 ^a^	2.28 ± 0.16 ^a^	2.28 ± 0.16 ^a^	2.28 ± 0.16 ^a^	2.28 ± 0.16 ^a^	2.28 ± 0.16 ^a^
10	6.55 ± 0.55 ^a^	4.82 ± 0.39 ^b^	5.62 ± 0.41 ^a^	5.27 ± 0.45 ^b^	5.03 ± 0.39 ^b^	4.70 ± 0.41 ^b^	4.36 ± 0.31 ^c^
20	11.64 ± 0.89 ^a^	8.78 ± 0.70 ^b^	10.27 ± 0.82 ^a^	9.46 ± 0.77 ^b^	9.05 ± 0.74 ^b^	8.69 ± 0.63 ^b^	8.02 ± 0.57 ^c^
30	19.10 ± 1.68 ^a^	16.23 ± 1.28 ^a^	17.66 ± 1.25 ^a^	16.22 ± 0.88 ^a^	16.46 ± 1.04 ^a^	15.96 ± 1.20 ^a^	14.10 ± 1.06 ^b^
60	32.92 ± 2.21 ^a^	29.67 ± 1.59 ^a^	31.26 ± 1.28 ^a^	30.86 ± 1.71 ^a^	30.35 ± 2.10 ^a^	29.21 ± 1.82 ^a^	28.02 ± 1.24 ^b^
120	47.93 ± 2.50 ^a^	43.07 ± 2.03 ^a^	46.35 ± 2.10 ^a^	43. 5 ± 2.44 ^a^	40.13 ± 2.33 ^b^	38.52 ± 2.09 ^b^	37.24 ± 1.84 ^c^
240	68.80 ± 2.33 ^a^	50.70 ± 2.89 ^d^	62.15 ± 3.31 ^b^	59.69 ± 3.68 ^c^	50.8 ± 3.46 ^d^	43.49 ± 2.20 ^e^	41.36 ± 1.78 ^f^

Means in a row (a–f across GSE level) followed by the same letter are not significantly different (*p* < 0.05).

**Table 4:** Correlation coefficients obtained by linear regression.

Y = f(X)	Correlation coefficient (*r*)	

	Convective heating	Microwave heating
TOTOX = f(TP)	−0.985	−0.986
CD = f(TP)	−0.966	−0.985
CT = f(TP)	−0.953	−0.981
PV = f(TP)	−0.972	−0.983
p-AV = f(TP)	−0.991	−0.986
IO = f(TP)	0.976	0.994

**Table 5:** Course of temperature for sunflower oil during convective and microwave heating.

Heating	Temperature (°C)						

	0	10 min	20 min	30 min	60 min	120 min	240 min
Convection oven	25.2	151.2	171.4	181.4	185.8	190.2	192.8
Microwave oven	26.4	144.6	157.6	178.2	181.4	185.6	190.2

## Abbreviations

GSE: grape seed extract
FRAP: ferric reducing antioxidant power
TP: total phenolic
PV: peroxide value
IO: inhibition of oil oxidation
p-AV: p-anisidine value
TOTOX value: total oxidation value
CD: conjugated dienes
CT: conjugated trienes
K 232: specific extinction value at 232 nm
K 268: specific extinction value at 268 nm
GAE: gallic acid equivalents
